# Mitochondrial Dysfunction and Oxidative Stress Caused by Cryopreservation in Reproductive Cells

**DOI:** 10.3390/antiox10030337

**Published:** 2021-02-24

**Authors:** Roberto Gualtieri, Guruprasad Kalthur, Vincenza Barbato, Maddalena Di Nardo, Satish Kumar Adiga, Riccardo Talevi

**Affiliations:** 1 Department of Biology, University of Naples “Federico II”, Complesso Universitario di Monte S. Angelo, Via Cinthia, 80126 Naples, Italy; barbato_vincenza@libero.it (V.B.); dinardomaddalena@gmail.com (M.D.N.); riccardo.talevi@unina.it (R.T.); 2 Department of Clinical Embryology, Kasturba Medical College, Manipal Academy of Higher Education, Manipal 576 104, India; guru.kalthur@manipal.edu (G.K.); satish.adiga@manipal.edu (S.K.A.); 3 Centre for Fertility Preservation, Kasturba Medical College, Manipal, Manipal Academy of Higher Education, Manipal 576 104, India

**Keywords:** mitochondria, cryopreservation, oxidative stress, sperm, oocyte, gonadal tissue

## Abstract

Mitochondria, fundamental organelles in cell metabolism, and ATP synthesis are responsible for generating reactive oxygen species (ROS), calcium homeostasis, and cell death. Mitochondria produce most ROS, and when levels exceed the antioxidant defenses, oxidative stress (OS) is generated. These changes may eventually impair the electron transport chain, resulting in decreased ATP synthesis, increased ROS production, altered mitochondrial membrane permeability, and disruption of calcium homeostasis. Mitochondria play a key role in the gamete competence to facilitate normal embryo development. However, iatrogenic factors in assisted reproductive technologies (ART) may affect their functional competence, leading to an abnormal reproductive outcome. Cryopreservation, a fundamental technology in ART, may compromise mitochondrial function leading to elevated intracellular OS that decreases sperm and oocytes’ competence and the dynamics of fertilization and embryo development. This article aims to review the role played by mitochondria and ROS in sperm and oocyte function and the close, biunivocal relationships between mitochondrial damage and ROS generation during cryopreservation of gametes and gonadal tissues in different species. Based on current literature, we propose tentative hypothesis of mechanisms involved in cryopreservation-associated mitochondrial dysfunction in gametes, and discuss the role played by antioxidants and other agents to retain the competence of cryopreserved reproductive cells and tissues.

## 1. Introduction

Historically, the first attempts to expose eukaryotic cells to low temperatures were performed on sperm by Spallanzani [1] but the first successful results on sperm cryopreservation had to wait till 1949 when Polge et al. [2] accidentally discovered the beneficial properties of glycerol as a cryoprotectant. Presently, cryopreservation of reproductive cells has become a necessary adjunct in human and animal assisted reproduction. It represents an invaluable tool for fertility preservation, gamete donation, preimplantation genetic diagnosis, reduction of multiple pregnancies, increase of cumulative pregnancy rates, as well as animal breeding and safeguarding germ plasm of endangered species. Successful species and cell-specific procedures for cryopreservation of sperm, oocytes, embryos, and gonadal tissue have been refined over the years. However, the viability and competence of reproductive cells are correlated with the quality and species of gametes and embryos, and with the extent of mitochondrial damage and oxidative stress (OS) induced by cryopreservation.

The primary reactive oxygen species (ROS) produced by cells are superoxide ions (O_2_^•−^), hydroxyl radicals (^•^OH) and hydrogen peroxide (H_2_O_2_). Different organelles and enzymes are involved in intracellular ROS production. Mitochondria are considered to be the major ROS producers in cells, even though several other organelles and enzymes contribute to such function [3]. The two major sources of intracellular ROS are plasma membrane NADPH oxidase and mitochondrial respiratory chain enzyme complexes. In addition, other enzymes in the cytosol, endoplasmic reticulum, and peroxisomes contribute to ROS production [4]. Although the exact contribution of mitochondria to total ROS production in different cells is still not completely understood, mitochondrial ROS are thought to regulate ROS production by non-mitochondrial sources [5].

Physiologically produced ROS at low levels by reproductive cells are essential in redox signaling, which is involved in sperm motility, capacitation, acrosome reaction, hyperactivation, transient sperm-oviduct adhesion, [6,7,8,9,10], and in oocyte maturation, fertilization, and embryo development [11,12,13,14,15,16,17]. However, when the ROS production greatly overwhelms the cell’s antioxidants defense system, a status of OS arises which can in turn affect the structure and function of proteins, lipids, and DNA, damaging the cell activity or leading to cell death. Helmut Sies first formulated the concept of OS in 1985 [18] as “a disturbance in the prooxidant-antioxidant balance in favor of the former” and then, after years of research on the physiological role of ROS in redox signaling, reformulated it as “an imbalance between oxidants and antioxidants in favor of the oxidants, leading to a disruption of redox signaling and control and/or molecular damage” [19]. Recently, the terms “oxidative eustress” and “oxidative distress” were introduced to more appropriately distinguish a mild OS that controls redox signaling in health (eustress) from a more intense OS that compromises redox signaling, damages biomolecules, and may lead to pathologies (distress) [20].

This manuscript aims to review the close and biunivocal relationships between mitochondrial damage and ROS generation during cryopreservation of gametes and gonadal tissues in different species. Here we investigated the role of mitochondria in the physiology of gametes and discussed how cryopreservation-induced mitochondrial dysfunction may impair their ability to fertilize and support embryo development. Furthermore, we emphasized the role played by mitochondrial antioxidants and other treatments to avoid such damage and to retain the competence of cryopreserved reproductive cells and tissues.

## 2. Mitochondria and ROS Production

Mitochondria have a pivotal role in the metabolism of nutrients and the consequent production of energy through the synthesis of adenosine triphosphate (ATP), and are responsible for ROS generation, homeostasis of calcium ions (Ca^2+^), and cell necrosis and apoptosis. They are delimitated by two membranes separated by an intermembrane space: an outer mitochondrial membrane (OMM) which is more permeable due to the presence of porins, and an inner mitochondrial membrane (IMM) representing the site of the electron transport chain (ETC), and oxidative phosphorylation (OXPHOS) which encloses the mitochondrial matrix [21]. The respiratory chain consists of five multi-protein complexes, from I to V, formed by at least 80 proteins, 13 of which are expressed by the mitochondrial genome. Complex I to IV constitute the ETC and the electrons transported along them finally reduce molecular oxygen (O_2_) to water. The electron transfer along the ETC is coupled with a transfer of protons in the intermembrane space through complex I, III, and IV, establishing an electrochemical gradient that enables the synthesis of ATP through complex V, the ATP synthase [22]. However, even under physiological conditions, the ETC leaks electrons leading to the univalent reduction of O_2_ to O_2_^•−^. About 0.4–4% of the O_2_ consumed by mitochondria is transformed into O_2_^•^− and then quickly dismutated by mitochondrial superoxide dismutase (SOD) into H_2_O_2_ [23]. Although complex I and III of the ETC have been classically considered to be the main sites of ROS production, recent studies demonstrated the presence of 12 sites associated with substrate catabolism and ETC that participate in H_2_O_2_ generation in mitochondria [20]. Specific sites acting as potential ROS sources depend on the species, tissue type, nature of substrate being oxidized, environmental O_2_ tension, the concentration of NADH electron donors, mitochondrial membrane potential (ΔΨm), and pH gradient [24,25,26,27].

Powerful scavenging systems for O_2_^•−^ and H_2_O_2_ regulate their levels in mitochondria and the cytosol but when their production rises too high, an oxidative distress status is established and the molecular oxidative damage cannot be repaired, causing a wide spectrum of different pathologies [26]. Although O_2_^•−^ has a high redox potential, it is not considered a strong oxidant because it does not permeate membranes and has a half-life of a few seconds. However, O_2_^•−^ can be rapidly dismutated into the powerful membrane permeant oxidant H_2_O_2_ by SOD or transformed into more reactive secondary radical species. Even at physiological pH, a low concentration of O_2_^•−^ can be protonated into the more damaging perhydroxyl radical (HO_2_^•^) that oxidates polyunsaturated fatty acids. Moreover, O_2_^•−^ can be transformed spontaneously into ^•^OH through the Haber–Weiss reaction or can generate the potent oxidant peroxynitrite (ONOO^−^) through its interaction with nitric oxide (NO) [28]. Among ROS, ^•^OH is considered the most powerful oxidant, and it can oxidate guanine into 8-oxoguanine, polyunsaturated fatty acids, amino acid residues of proteins or DNA bases and deoxyribose [28].

Under physiological conditions, i.e., during eustress, H_2_O_2_ is considered to be the major ROS involved in redox regulation. However, supraphysiological H_2_O_2_ concentrations damage biomolecules leading to oxidative distress [20]. Although H_2_O_2_ easily crosses the cell membranes, it reacts poorly with biomolecules and the most damaging effects of H_2_O_2_ arise from its metal-catalyzed conversion into ^•^OH by the Fenton and Haber–Weiss reactions [28].

Mitochondrial DNA and the ETC complexes are considered to be the primary targets of free radical attack and this may increase O_2_^•−^ production due to the altered function of ETC proteins or to the decreased expression of critical ETC proteins encoded by the mitochondrial genome [29,30].

Mitochondria are also central in regulating the Ca^2+^ homeostasis that plays a crucial role in cell physiology and pathology. Mitochondrial Ca^2+^ uptake, driven by ΔΨm, regulates the levels and spatio-temporal patterns of intracellular Ca^2+^ signals [31]. Both Ca^2+^ and ROS act as second messengers in cell signaling and interact with each other bidirectionally. In fact, ROS can regulate intracellular Ca^2+^, and increased Ca^2+^ levels can activate ROS generation [32]. Ca^2+^ ions freely permeate through the OMM and enter the mitochondrial matrix via the Ca^2+^ uniporter located in the IMM [33].

Moreover, mitochondrial Ca^2+^ and ROS [34] are the two main activators of a large conductance channel, the mitochondrial permeability transition pore (mPTP) whose opening increases the permeability of IMM to solutes up to1500 Da leading to depolarization, Ca^2+^ and ROS release [5]. Transient short-term openings of mPTP are thought to fulfill physiological functions through release of Ca^2+^ and ROS, whereas prolonged openings lead to rapid collapse of the ΔΨm, the consequent ATP loss, as well as osmotic shock causing the rupture of the OMM. These events finally result in necrosis [35,36,37] but can also cause the release of the mitochondrial pro-apoptotic factors, cytochrome C, apoptosis-inducing factor, activator of caspases, and endonuclease G, leading to apoptosis [38,39,40,41].

## 3. Roles of Sperm Mitochondria

Mammalian sperm generally have approximately 50 to 80 mitochondria, tightly wrapped to form a mitochondrial sheath in the midpiece of the flagellum [42,43]. Mitochondria are essential in several sperm functions and through ATP production, they regulate spermatogenesis, capacitation, induction of acrosome reaction, oocyte fusion, and fertilization [44,45,46]. Several reports indicate that motility and fertility of human sperm is closely correlated with mitochondrial function and these organelles have been indicated as biomarkers of sperm quality in several species [47,48,49]. Mitochondrial membrane potential and respiratory efficiency have been positively correlated with human sperm motility [50,51] and several studies proposed that assessment of ΔΨm predicts the sperm fertilization competence both in natural conception and in vitro fertilization (IVF) [52,53,54,55]. Recently, high ΔΨm has been suggested to be necessary for acrosin activity, induction of acrosome reaction and maintenance of chromatin integrity of human sperm [56].

Mutations of sperm mtDNA have been associated with decreased sperm quality and may impact the efficiency of OXPHOS as several ETC proteins are encoded by mtDNA. In particular, a lack of efficient mtDNA repair mechanisms in sperm [57] has been linked to the occurrence of a more rapid accumulation of mutations than in somatic cells [45]. Sperm mtDNA mutations found in asthenozoospermic and oligoasthenozoospermic men have been reported to compromise semen quality [58,59]. Moreover, an inverse relationship has been reported between sperm mtDNA copy number, and semen quality and fertility [60,61,62], although the outcome of intracytoplasmic sperm injection (ICSI) was not affected [63].

**Figure 1:**
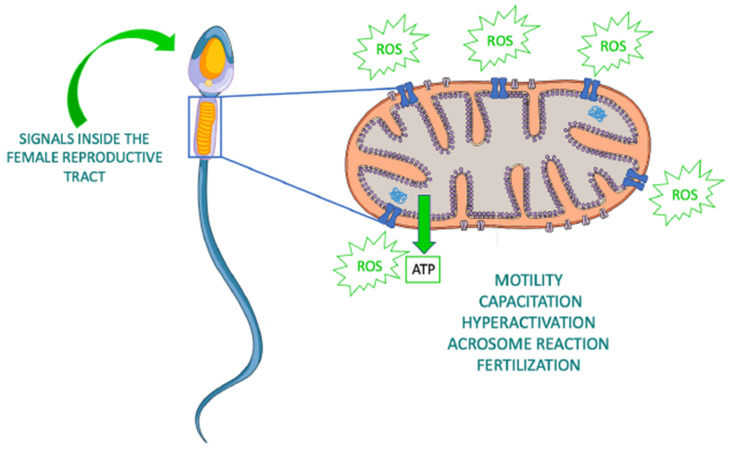
Roles of sperm mitochondria in health. Production of ATP and ROS by sperm mitochondria is involved in several sperm functions. Modified from https://www.vecteezy.com/vector-art/1434164-human-sperm-or-spermatozoa-cell-structure (accessed on 28 December 2020).

Mitochondria also represent the major source of ROS and reactive nitrogen species (RNS) in sperm and have a central role in redox signaling that drives fundamental events in the sperm life such as the activation of motility, hyperactivation, capacitation, acrosome reaction, and fertilization [64] (Figure 1). However, oxidative and nitrosative stress caused by excessive ROS and RNS production respectively, have been identified as the major cause of male infertility [65]. In fact, spermatozoa are extremely prone to ROS-induced damage as they contain modest antioxidant defenses and a high content of oxidizable substrates [66,67,68]. In particular, the sperm plasma membrane is rich in polyunsaturated fatty acids that regulate membrane fluidity, but also represent preferential substrates for ROS attack. This, in turn, triggers a lipid peroxidation cascade and the consequent generation of highly reactive lipid aldehydes that covalently bind to ETC proteins reinforcing the production of mitochondrial ROS and compromising both the competence and the DNA integrity of the spermatozoa [69,70,71].

## 4. Effects of Sperm Cryopreservation on Mitochondria and Oxidative Stress

Sperm cryopreservation has become a routine procedure in human ART and is a common method for preserving and transporting genetic material in most domestic species. Nonetheless, an impressive plethora of evidence on human and domestic animals indicates that sperm cryopreservation decreases the number of viable sperm and can affect the functions of surviving cells by impairing their motility, mitochondrial activity, chromatin integrity, and reproductive potential [72,73,74,75,76]. Slow-freezing procedures, the first historically developed techniques to successfully cryopreserve spermatozoa, remain the most commonly used techniques [77], probably because the high sperm number generally present in ejaculates make survival rates acceptable. On the other hand, spermatozoa in poor-quality ejaculates are more prone to cryoinjuries and the possibility to recover viable sperm post-thawing has been reported to be difficult in severely oligozoospermic patients [78]. Adverse effects of cryopreservation on sperm motility, DNA integrity, and sperm competence were reported especially in subfertile and infertile men [79,80,81]. OS is regarded as one of the main factors underlying both male infertility and the reduced survival and competence of sperm observed after cryopreservation. This is particularly concerning as, at least in non-human mammals, sperm OS has been correlated with a significant reduction in fertilization rates and in vitro embryo development [82,83,84,85]. Hence, it is important to look for causes and remedies of cryopreservation-associated sperm damage. This may lead to refine cryopreservation procedures through pre- or post-treatment with antioxidants to improve the survival and competence of sperm of infertile patients or those of animal species that scarcely tolerate the stress imposed by cryopreservation. DNA damage and loss of mitochondrial function have been commonly associated with cryopreservation of mammalian sperm and together with ROS levels represent the more frequently assessed endpoints in studies aimed to improve the outcome of the procedure [86]. Changes of mitochondrial membrane fluidity occurring during cryopreservation have been suggested to raise ΔΨm and to induce the release of ROS. Released ROS, in turn, cause DNA damage leading to single/double-strand DNA breaks [87].

Detailed studies on the relevance of mitochondrial damage on the decreased performance of frozen–thawed mammalian sperm are available in domestic animals. In ovine spermatozoa, mitochondrial cryoinjuries reduce the ability of frozen–thawed sperm to migrate through the cervix and survive in the female reproductive tract [88]. Opening of the mPTP seems to be involved in sperm cryodamage in different species. The first indirect evidence of such an involvement has been reported in the stallion in which supplementation of sperm freezing media with bongkrekic acid, an inhibitor of mPTP opening, reduced active caspases and increased ΔΨm in thawed sperm [89]. Recent studies on cryopreserved bull sperm confirmed that mitochondrial dysfunction is due to the opening of the mitochondrial permeability transition pore in response to intracellular Ca^2+^ increases and this was associated with loss of ΔΨm, decreased ATP content, increased ROS levels, and deterioration of plasma membrane integrity [90]. The involvement of mPTP opening in sperm cryopreservation has been confirmed in ram and the ability of the mitochondrial antioxidant melatonin to prevent this effect was carefully assessed [91,92]. Melatonin is an antioxidant localized and mainly synthesized in mitochondria that is actively accumulated via mitochondrial melatonin transporter. Melatonin maintains ΔΨm and preserves mitochondrial functions through scavenging of both ROS and RNS, inhibition of mPTP opening, and it also acts as a signaling molecule that up-regulate the expression of antioxidant enzymes and stress responsive genes [93]. On this basis, melatonin has been used to prevent cryoinjuries related to mitochondria and OS during cryopreservation of human and domestic animal sperm. In 2011, Succu et al., [94] demonstrated that the addition of melatonin to ram semen freezing extender led to higher viability, motility, intracellular ATP levels, and DNA integrity compared to semen frozen in non-supplemented extenders. In addition, melatonin-treated sperm had better performance in IVF compared to control frozen–thawed sperm, showing higher fertilization rates and a reduction of time to the first embryonic division [94] likely due to decreased sperm DNA damage and a faster repair in the zygote. More recent studies show that melatonin supplementation of ram sperm freezing media inhibits mPTP opening, leading to improvements in key OXPHOS enzymes, oxygen consumption, ATP synthesis, and sperm viability, motility and kinetics [91,92]. Melatonin supplementation during the 4 °C equilibration period was most effective for inhibiting mPTP opening, decreasing the enzymatic activity of Cyclophilin D (a key mediator of mPTP opening), and increasing plasma membrane integrity, ΔΨm, and mitochondrial Cyt C concentration. Moreover, melatonin-treated frozen–thawed sperm produced higher hatching blastocysts and pregnancy rates after IVF and artificial insemination respectively [92]. Overall, considerable direct evidence in domestic animals suggest mPTP opening as a common denominator of sperm cryodamage.

In human sperm, supplementation of freezing media with melatonin increased sperm viability and membrane integrity, decreasing ROS and lipid peroxidation. Moreover, melatonin increased mRNA levels of the transcription factor NF-E2-related factor-2 and its downstream genes which play a critical role in the defense against OS, and up-regulated the expression of the antiapoptotic genes Bcl-2 and heat shock protein 90 (HSP90), which confers resistance to stressors in frozen–thawed sperm [95]. Najafi et al. showed that supplementation of human sperm freezing media with melatonin increases AKT phosphorylation, and decreases ROS, caspase-3 activity, and both dead and apoptotic-like sperm via the PI3K/AKT signaling pathway [96]. A more recent study indicated that supplementation of human sperm freezing solution with melatonin, followed by thawing in the presence of both melatonin and caffeine, prevented the loss of progressive motility and mitochondrial activity in sperm of normozoospermic men. However, this treatment failed to prevent the increase of DNA fragmentation and ROS production compared to fresh samples [97].

**Figure 2:**
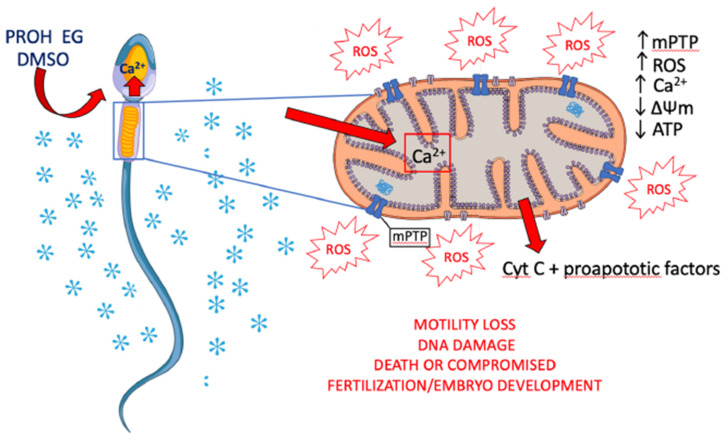
Possible mechanisms of sperm mitochondrial dysfunction associated with cryopreservation. Exposure to permeant cryoprotectants cause intracellular Ca^2+^ increase. Prolonged openings of mPTP due to Ca^2+^ overload trigger ROS and Ca^2+^ release, loss of ΔΨm, decreased ATP content, and release of Cyt C. Such events may culminate in DNA damage and apoptosis. Depending on the extent of mitochondrial damage, the sperm can die or survive to cryopreservation. Oocytes fertilized by survived, DNA damaged sperm, could hesitate into a compromised embryo development. PROH, propanediol; EG, ethylene glycol; DMSO, dimethyl sulfoxide. Modified from https://www.vecteezy.com/vector-art/1434164-human-sperm-or-spermatozoa-cell-structure (accessed on 28 December 2020).

Based on literature data on cryopreservation-associated mitochondrial dysfunction in sperm of different species, it can be speculated that cryoprotectant-induced Ca2+ overload might induce prolonged openings of mPTP leading to ROS and Ca2+ release, loss of ΔΨm, decreased ATP content, and release of Cyt C (Figure 2).

Mitochondria-targeted antioxidants have been generally designed through a combination of antioxidants with the lipophilic cation triphenylphosphonium (TPP) which drive their massive accumulation in a ΔΨm dependent manner to extinguish oxidative stress in pathological mitochondria [98]. Supplementation of cell permeable ROS scavenger MitoTEMPO, a combination of the antioxidant piperidine nitroxide TEMPO and TPP [99] in commercial sperm freezing media has improved post-thaw sperm motility, viability, ΔΨm, antioxidant enzymes activities, and decreased ROS levels and malondialdehyde (MDA) content in human normozoospermic and asthenozoospermic ejaculates [100,101].

MitoQ or mitoquinone is one of the most well characterized mitochondrial-targeted antioxidants and consists of oxidized CoQ10 moiety conjugated to TPP to facilitate accumulation within the mitochondria [102]. MitoQ significantly decreased ROS production, as well as lipid peroxidation, and increased post-thaw viability in yellow catfish sperm [103]. Addition of MitoQ to the freezing extender has been reported to improve the quality of human sperm by decreasing ROS and MDA levels and preventing ΔΨm loss in thawed sperm [104].

Elamipretide, also known as SS-31, a water-soluble and permeant tetrapeptide that selectively binds cardiolipin, is the first cardiolipin-protective compound used as a therapeutic agent in a variety of mitochondrial diseases [105]. Cardiolipin, a phospholipid exclusively localized on IMM, plays an important role in cristae formation and the assembly of ETC complexes into supercomplexes for optimal OXPHOS and is considered a platform for initiation of apoptosis [105]. Oxidized cardiolipin transforms cytochrome c into a peroxidase leading to further cardiolipin oxidation, release of cytochrome c and apoptosis [106]. Cardiolipin is present in the mitochondria in human and rat testis and is the substrate for the synthesis of a fully saturated form of cardiolipin found exclusively in extramitochondrial locations such as the acrosome membranes [107,108,109]. Interestingly, a recent study demonstrated that Elamipretide was able to protect cryopreserved human sperm improving their motility, viability, acrosomal function, membrane and chromatin integrity, ΔΨm, antioxidants enzyme activities, and preventing the increase of ROS and MDA associated with cryopreservation [110]. The fact that acrosomal function of frozen–thawed sperm was improved could indicate that Elamipretide protects both mitochondrial cardiolipin and the newly discovered acrosomal saturated cardiolipin. Incubation with micelles containing a mitochondrial-like mixture of glycerolphospholipids, including the key precursor of cardiolipin, phosphatidylglycerol, improves the viability, motility and the resistance to oxidation of human sperm [111].

Among the non-enzymatic mitochondrial ROS scavengers, a major role is played by the lipid-soluble antioxidant vitamin E, which reacts with the peroxyl radicals faster than the molecules of polyunsaturated fatty acids, and in doing so, protects membranes from excessive oxidative damage [112]. Several studies evaluating the ability of alfa-tocopherol, an active form of vitamin E, to protect mammalian sperm from OS associated with cryopreservation showed decrease of ROS and lipid peroxidation, and improvements of post-thaw motility, ΔΨm and DNA integrity [113,114,115,116,117,118,119]. Recently, both alpha and gamma tocopherol have been identified in human seminal plasma and the supplementation of freezing media with gamma tocopherol induced a higher post-thaw human sperm viability and motility than alpha-tocopherol [120]. Interestingly, the use of nano-emulsions containing vitamin E to protect it from oxidation has been demonstrated to protect acrosome integrity and mitochondrial activity of frozen–thawed red deer sperm, preventing sperm lipoperoxidation and reducing ROS production [121].

L-carnitine plays a crucial role in sperm health by facilitating the transport of activated fatty acids across the IMM, so that they can be broken down through beta-oxidation to produce ATP [122]. Zhang et al., showed that supplementation of cryopreservation media with l-carnitine reduced spermatozoa cryodamage in both asthenozoospermic and normozoospermic human semen samples [123].

## 5. Roles of Oocyte Mitochondria

Mitochondria are inherited exclusively from the cytoplasm of the fertilized oocyte as sperm mitochondria are actively excluded from the zygote to limit heteroplasmy and its detrimental consequences on development and health of the offspring [124]. Due to the absence of protective histones, low DNA repair ability, and its proximity to the ROS producing ETC, mitochondrial DNA (mtDNA) is 10- to 50-fold more prone to mutations than nuclear DNA [125].

During embryo development, a bottleneck positioned between the primordial germ cells and the primary oocyte dramatically reduces mtDNA copy numbers, preventing the accumulation of mutant mtDNA in the maternal germ line and heteroplasmy in the offspring [126]. During human folliculogenesis, mtDNA copy number increases from about 500 copies in primary oocytes to 150,000–700,000 copies in mature oocytes [127]. The mature human oocyte is the richest cell in terms of mtDNA content, which is required to acquire competence for fertilization and for the embryo to reach the blastocyst stage [128]. Oocyte’s mitochondrion is considered immature and it has a round or oval shape, few cristae, a dense matrix and generally one or two mtDNA genomes [129]. As mtDNA replication ceases until later stages of pre- or post-implantation development, each embryo cleavage halves the number of mitochondria per cell until mitochondrial replication is resumed [130,131]. Therefore, until this stage the embryo relies exclusively on the mitochondrial pool inherited from the oocyte. At this time, a transition occurs, and the mitochondria acquire an elongated shape, a lighter matrix and numerous transverse cristae and increase their oxygen consumption and OXPHOS [132].

Despite their immature appearance, oocyte mitochondria are active in OXPHOS and their ATP production is required for several essential events such as oocyte maturation, spindle assembly, polar body extrusion, chromosome segregation, fertilization, and embryo development [14,130].

Mitochondrial membrane potential, OXPHOS, and oocyte ATP levels increase during oocyte maturation [133,134,135,136]. Oocytes ATP levels have been positively correlated with fertilization rates and embryo development and ATP content ≥ 2 pmol/oocyte is needed to support normal embryo development [134,137,138].

**Figure 3:**
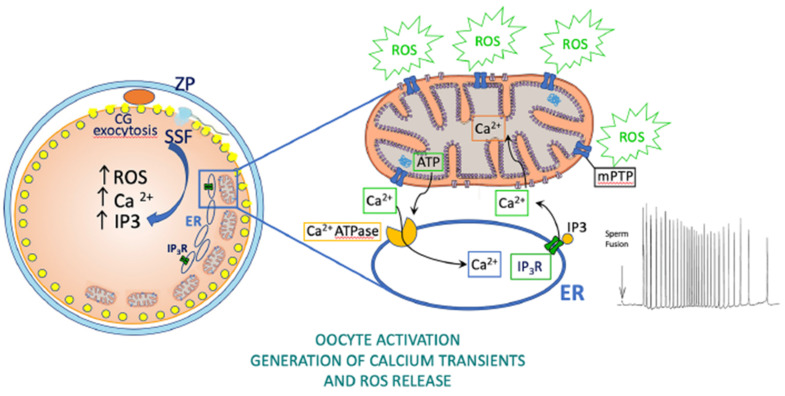
Roles of oocyte mitochondria in health. Production of ATP and ROS by oocyte mitochondria is involved in fertilization and embryo development. Upon gamete fusion, diffusion of a sperm soluble factor (SSF) into the oocyte cytosol generates IP3 which binds endoplasmic reticulum (ER) IP3 receptors (IP3R) causing cytosolic Ca^2+^ oscillations. Cytosolic Ca^2+^ rise drives numerous events among which the exocytosis of cortical granules (CG) and the consequent hardening and polyspermy block of the zona pellucida (ZP). Ca^2+^ potentiates the mitochondrial production of ATP which is needed for Ca^2+^ reuptake into the endoplasmic reticulum through Ca^2+^ ATPase allowing the recovery of cytosolic Ca^2+^ levels after each transient. Evidence in oocytes from different species indicates that sperm-induced Ca^2+^ rise triggers a release of ROS from mitochondria which is required for embryo development.

Oocyte mitochondria are involved in Ca^2+^ homeostasis and Ca^2+^ oscillations at fertilization and these, in turn, regulate correct embryo development [14,139]. In fact, sperm-triggered cytosolic Ca^2+^ transients directly stimulate mitochondrial activity, and the latter is absolutely required to maintain basal cytosolic Ca^2+^ levels in the unfertilized oocyte and to recover the basal level after each Ca^2+^ transient at fertilization [139]. Subplasmalemmal highly polarized mitochondria of mammalian oocytes have been suggested as a prerequisite for fertilization competence [140]. Mature mouse and human oocytes with a reduced complement of subplasmalemmal mitochondria cannot be penetrated by sperm [135,140]. In addition, evidence in different species, from marine invertebrates to mice and bovines, indicates that sperm penetration drives a burst of H_2_O_2_ production and oxygen consumption suggesting that mitochondrial activity is stimulated by Ca^2+^ increases around the time of sperm penetration [11,12,14,141]. Elegant experiments on transgenic *Xenopus laevis* oocytes expressing the H_2_O_2_ indicator HyPer demonstrated that intracellular Ca^2+^ waves are necessary and sufficient to induce a rapid increase of mitochondrial H_2_O_2_ production at fertilization which oscillate with each cell division during embryo development. Moreover, ROS oscillations have been suggested to modulate the cell cycle through phosphatase Cdc25C and attenuation of mitochondrial ROS production blocked the cell cycle [17] (Figure 3).

The pivotal role of mitochondria, and their dysfunctions, as determinants of oocyte quality and embryo developmental potential has been clearly recognized in patients of advanced reproductive age, or with obesity, diabetes, and other metabolic disorders [142,143]. Reproductive aging has been associated with reduced ΔΨm and ATP production, altered gene expression, increased ROS generation and accumulation of mtDNA mutations both in human and in animal models [144,145,146,147] and these can in turn induce spindle damage, chromosome misalignment and aneuploidy [148,149]. A decreased expression of the mitochondrial antioxidant genes, peroxiredoxin 3 and thioredoxin 2, has been reported in aged mice oocytes [150]. Aging also affects mtDNA copy number and ATP levels in different species and a decreased copy number has been reported in older than in younger oocytes or embryos [151,152,153]. The determination of mtDNA copy number in oocyte granulosa cells or in blastocyst’s trophectoderm biopsies has been recently suggested as a method to predict the embryo quality [154]. Graded mitochondrial injury in mouse oocytes suggested that the extent of mitochondrial injury determines whether the oocyte dies or the resulting embryo will proceed through the early embryo development before death occurs [155].

Several approaches have been developed for replacing dysfunctional mitochondria of poor-quality oocytes with healthy mitochondria to restore oocyte quality [156]. These approaches have also been used as a therapy to eliminate mitochondrial mutations and prevent the transmission of mtDNA disorders [157,158,159]. Although 25 babies were born through injection of donor oocyte cytoplasm so far, this procedure has been discontinued for concerns of heteroplasmy or transmission of mitochondrial diseases from the donor [160,161,162]. New procedures relying on the transfer of autologous sources of mitochondria from the patient’s granulosa or ovarian stem/precursor cells to ensure the provision of high-quality homologous mitochondria with intact and homoplasmic mtDNA could represent promising tools to restore oocyte quality [163].

Taken together, this evidence emphasizes the essential role of mitochondria as determinants of oocyte quality and any insult disturbing mitochondrial replication and/or functioning might potentially impair oocyte competence.

## 6. Effects of Oocyte Cryopreservation on Mitochondria, Oxidative Stress, Fertilization, and Embryo Development

During the last two decades, great efforts were devoted to the development of successful protocols for oocyte cryopreservation to avoid the ethical and legal problems connected to embryo cryopreservation, to preserve female fertility, and to manage oocyte donation [164,165,166]. Although the first births from human cryopreserved oocytes were reported more than 30 years ago [167,168], the initial slow-freezing procedures adopted, and their variations had a very limited success rate and oocyte cryopreservation did not spread worldwide until Kuwayama and collaborators [169] proposed vitrification as an elective strategy to maintain the developmental competence of cryopreserved oocytes. Oocyte vitrification has become a routine technology in human ART and fertility preservation programs and is no longer considered experimental [170]. Initial attempts to cryopreserve oocytes were profoundly limited by their imperfect methodologies, with the consequence being poor clinical outcomes. In fact, several studies demonstrated that during slow-freezing, cellular and molecular alterations associated with osmotic forces produced during oocyte dehydration–rehydration cycles or by cryoprotectants may affect the molecular architecture of the meiotic spindle and chromosomes, as well as the distribution and activity of other cytoplasmic components [171]. On the other hand, oocyte vitrification, in which intracellular and extracellular fluid is transformed from a liquid into a glassy amorphous solid state, retains the distribution of molecules and ions and reduces damages to oocytes, exhibiting a better clinical outcome than that of slow-freezing [172,173,174,175].

Studies on reproductive cells from different species have indicated that cryopreservation induces alterations and/or damages to the mitochondria [176,177,178]. A more detailed comprehension of the effects of cryopreservation on mitochondrial function and OS has been achieved for sperm of different species, whereas only a partial and fragmentary knowledge is available for oocytes.

Studies have shown that cryopreservation by slow-freezing process could alter oocyte ΔΨm but not inducing early apoptotic response [179,180]. Similarly, vitrification of mouse oocytes can alter mitochondrial distribution and reduce the ΔΨm [181] which was also found to be age related [182]. On the other hand, it was found that oxidation of the intracellular redox potential by vitrification was independent of age [183]. Vitrification significantly reduces the ATP content in bovine, human, rabbit, murine, and porcine oocytes [184,185]. It has been suggested that the rapid entry of cryoprotectants into the oocytes can cause irreversible damage to cell organelles, including mitochondria [186]. Since vitrification uses higher concentrations of cryoprotectants, and a mature oocyte’s cytoplasm is normally more hydrated than in embryos, the extent of impact on cell architecture could be higher. This has been supported by the fact that vitrification can severely damage mitochondria, alter the expression of mitochondria functional genes in bovine mature oocytes when cryopreserved at immature stage and these changes are found to be related to the concentrations of cryoprotectants used [187]. Mouse prepubertal oocytes cryopreserved after in vitro maturation (IVM) have shown lower ΔΨm than prepubertal oocytes cryopreserved at germinal vesicle stage [188]. In contrast, no significant alteration in mitochondrial distribution was observed between human oocytes cryopreserved before and after IVM [189]. Interestingly, vitrification-warming of human mature oocytes resulted in either a transient reduction of the ΔΨm [190] or failed to demonstrate any changes in ΔΨm and intracellular ROS levels [183]. In mouse oocytes, all the permeant cryoprotectants used in slow-freezing and vitrification cause intracellular Ca^2+^ rises comparable to the initial increase triggered at fertilization. More specifically, both ethylene glycol and propanediol vehiculate Ca^2+^ from the external medium, propanediol exerting a more powerful and long-lasting Ca^2+^ rise, whereas dimethyl sulfoxide (DMSO) releases Ca^2+^ from internal stores [191,192]. By fact, use of Ca^2+^-free media, extracellular or intracellular Ca^2+^ chelation has been reported to improve oocyte vitrification in mouse, ovine, bovine [94,185,191,192,193,194].

Bovine oocyte vitrification, especially with DMSO, has been showed to trigger the release of Ca^2+^ from the endoplasmic reticulum leading to abnormally increased cytosolic and mitochondrial Ca^2+^ levels. This was associated with cortical granules exocytosis, decreased ΔΨm, ATP content, increased TUNEL positivity, and reduced cleavage and blastocysts rates. Treatment with the intracellular Ca^2+^ chelator BAPTA and the mitochondrial Ca^2+^ uniporter inhibitor ruthenium red during vitrification decreased cytosolic and mitochondrial Ca^2+^ respectively. Moreover, it also improved cleavage and blastocysts rates, as well as the expression of genes related to blastocysts apoptosis and implantation, to levels similar to fresh oocytes. The decreased ΔΨm and ATP content of vitrified bovine oocytes was suggested to derive from mitochondrial membrane permeability transition in response to mitochondrial Ca^2+^ overload [185].

Mitochondrial Ca^2+^ overload and OS can drive cell damage and death through opening of the mPTP that affects the ΔΨm, Ca^2+^ homeostasis, ATP and ROS production [195,196,197,198]. Evidence indicates that mPTP opening is a cause of damage during cryopreservation of sperm (see above), whereas such an involvement has poorly been investigated in oocytes. However, pretreatment of mouse oocytes with the specific inhibitor of mPTP, cyclosporine A, before H_2_O_2_ exposure, has been reported to prevent ΔΨm loss and ATP reduction, thus increasing the developmental potential of oocytes [155]. In addition, pretreatment of bovine oocytes with cyclosporine A before vitrification prevented the increase of ROS in oocytes, and increased the blastocyst rate and ATP content of blastocysts derived from parthenogenetically activated vitrified oocytes [184].

Oocyte mitochondria play a key role in the generation of intracellular Ca^2+^ transients at fertilization and their alterations may exert long-term detrimental effects on pre- and post-implantation development [199,200,201]. Gualtieri et al. suggested that loss of ΔΨm and mitochondria ultrastructural degeneration found in human oocytes cryopreserved through the 0.3-M sucrose slow-freezing method could compromise oocyte Ca^2+^ signaling and developmental competence [180], contributing to the reduced clinical outcome of this procedure [202,203,204]. Analysis of intracellular Ca^2+^ response to ionomycin in cryopreserved human oocytes showed a marked delay in recovery of Ca^2+^ basal levels in slow-frozen and not in vitrified oocytes. These findings agree with the lower mitochondrial damage found in vitrified versus slow-frozen oocytes [205]. Although a direct relationship with mitochondrial damage is lacking, vitrification of mouse oocytes has also been found to affect Ca^2+^ signaling [206,207].

**Figure 4:**
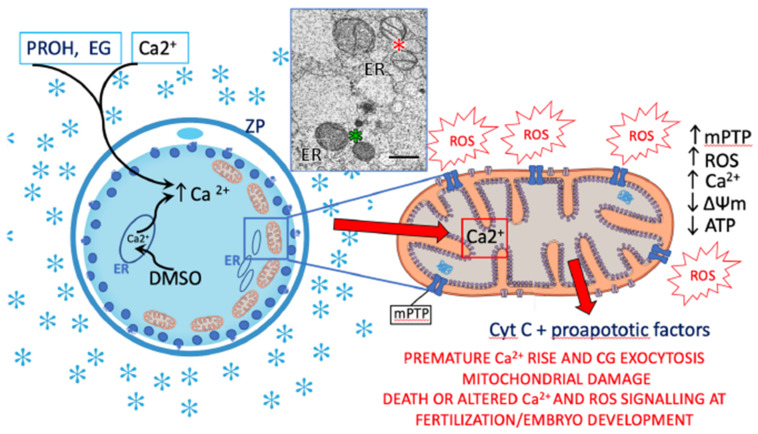
Possible mechanisms of oocyte mitochondrial disfunctions associated with cryopreservation. Exposure to permeant cryoprotectants causes intracellular Ca^2+^ increase. Propanediol (PROH) and ethylene glycol (EG) vehiculate external Ca^2+^, whereas dimethyl sulfoxide (DMSO) triggers Ca^2+^ release from the endoplasmic reticulum (ER). Premature Ca^2+^ rise causes partial cortical granule (CG) exocytosis and zona pellucida (ZP) hardening. Prolonged openings of mPTP due to Ca^2+^ overload trigger ROS and Ca^2+^ release, loss of ΔΨm, decreased ATP content, and release of Cyt C. Such events may culminate in DNA damage and apoptosis. The extent of mitochondrial injury in cryopreserved oocytes determines whether the oocyte dies or survives. Substantial mitochondrial damage might lead to altered Ca^2+^ and ROS signaling at fertilization, and compromised embryo development. Inset, intact (green asterisk) and damaged (red asterisk) mitochondria in a human oocyte cryopreserved through slow cooling (bar = 0.5 μm).

Several studies demonstrate clear links among oocyte cryopreservation, mitochondrial damage, OS, and embryo development in animal models. Decrease in ΔΨm and increase in ROS levels were reported in vitrified mouse [180] and porcine oocytes where, in addition, mitochondria ultrastructural damage, decrease of ATP content and dysregulation of mitochondria and apoptosis-related genes were also reported [208,209]. A hypothetical mechanism of mitochondrial dysfunction associated with oocyte cryopreservation is proposed in Figure 4.

Oocyte cryopreservation-induced sub-lethal injuries such as OS induced DNA damage, altered metabolism, transcription and translation abnormalities may not be detectable morphologically but a significant number of such oocytes eventually may fail to get fertilized [210]. From the clinical perspective, it is important to know that only ~2% of human oocytes cryopreserved through slow-freezing can develop to term while majority of them survive cryopreservation [211]. Mitochondrial dysfunction in oocytes has been correlated with embryo arrest in vitro [155]. Slow-freezing and vitrification similarly affect the genes involved in oxidation-reduction pathway in cleavage stage preimplantation embryos derived from cryopreserved mouse oocytes [212,213].

Abnormal expression of apoptotic and mitochondria-related genes in bovine embryos derived from the vitrified oocytes were associated with vitrification temperature and the concentration of cryoprotectants used [187]. Furthermore, the disruptions in the embryonic genome activation due to oocyte cryoinjury can impair several essential processes in embryos including mitochondrial function [212]. Though mtDNA content in human blastocysts was not affected by the oocyte vitrification [214], it should be noted that the copy number of mtDNA alone cannot reflect the mitochondrial turnover hence, further studies are required to better understand the mitochondrial integrity between fresh and vitrified sibling oocytes in human.

Mammalian oocytes have large cytoplasm with abundant mitochondria in the ooplasm which can experience structural and functional damage during the cryopreservation process. Therefore, reduction in viability and post-thaw developmental competence can be correlated with the extent of mitochondrial damage in oocytes. A minimum of 2 to 4 h may be required for recovery of mitochondrial function following vitrification in porcine oocytes [215]. Antioxidants such as melatonin, *N*-acetyl cysteine and, combination of ascorbic acid and rosmarinic acid have shown to improve the cryosurvival of the mouse oocytes and enhance their developmental potential [216,217,218]. Alpha-tocopherol treatment during recovery culture improved development of embryos generated from vitrified-warmed bovine oocytes [219]. Moawad et al., demonstrated that l-carnitine supplementation during vitrification of Germinal vesicle (GV) stage oocytes reduced the mitochondrial aggregation and improve the distribution pattern along with improved spindle organization and nuclear maturation of mouse oocytes subjected to IVM [220]. Similarly, supplementation of glutathione donor, glutathione ethyl ester (GEE), in cryopreservation medium has shown to improve the cryotolerance of mouse oocytes by preventing the loss of mitochondrial function [221] and reducing the oxidative stress in bovine oocytes [222]. Furthermore, Li et al. have shown that pre-incubation of mouse oocytes with GEE enhances the developmental competence of vitrified oocytes by preventing the mitochondrial damage and altering the expression of pro- and anti-apoptotic genes [223].

## 7. Effects of Gonadal Tissue Cryopreservation on Mitochondria and Oxidative Stress

Gonadal tissue cryopreservation has gained attention in recent years due to its application in fertility preservation field [224]. Various protocols have been followed to store ovarian and testicular tissues from prepubertal and young adults. In either case, due to the complexity of the tissue, designing an optimal cryopreservation protocol is technically challenging. Even though preserving the integrity of the germ cells is the key interest in such situation, retaining the architecture of extracellular matrix, communication between different cell types and preserving the organelle function are very critical. Mitochondria being the major source of OS, is expected to undergo functional changes after cryopreservation process.

Mitochondria are considered to be the most sensitive organelles in oocytes [225]. Altered distribution patterns, and ultrastructural damage of mitochondria was observed in oocytes of frozen–thawed human ovarian tissue and of vitrified mouse ovarian tissue [226,227]. Mitochondrial damage in human ovarian tissues, specifically in oocytes has been reported during freeze-thaw process by earlier studies which may have a significant effect on follicle growth [228,229]. However, conventional liquid nitrogen and ultra-rapid slush nitrogen vitrification did not affect mitochondrial ultrastructure [230] even though the expression analysis of genes involved in OS, hypoxia, osmotic stress, and cell death, demonstrated a markedly higher dysregulation in conventional versus ultra-rapid vitrification [231]. Ultrastructural assessment of mitochondrial organization may be considered to be a parameter to evaluate the efficiency of cryopreservation protocol; however, the mitochondrial dysfunction need not be always associated with morphological changes. Cryopreservation of mouse ovarian tissue by the slow-freezing technique resulted in a significantly higher level of ROS and DNA fragmentation in oocytes in comparison to vitrification [232]. Increased level of free Ca^2+^ and reduction in ATP level was observed in oocytes of vitrified mouse ovaries [233]. Moreover, vitrification of rat ovaries has been reported to increase ROS, malondialdehyde and nitric oxide levels, and the addition of the mitochondrial antioxidant melatonin to the vitrification solutions protected follicular integrity preventing such effects and increasing the activities of glutathione peroxidases, glutathione, catalase, and superoxide dismutase [234].

Overall, mitochondrial changes in oocytes and stromal cells are a common observation in ovarian tissue cryopreservation suggesting there is still scope of improvement in designing the protocol.

The impact of cryopreservation on mitochondria and OS in testicular tissue has been poorly addressed. However, cryopreservation has been reported to induce OS in mouse, bovine, and human testicular tissue. In the mouse, an increased ROS production was reported in frozen–thawed versus fresh tissue and vitamin E and not GSH supplementation of culture media after thawing decreased ROS levels and enhanced sperm differentiation in vitro [235]. In bovine, testicular tissue cryopreservation decreased total antioxidant, catalase and superoxide dismutase activities, and led to an increase of malondialdehyde content. Addition of 15% trehalose reduced the OS and improved the cryoprotective effects [236].

Although Moubasher et al. did not find increased malondialdehyde production in frozen–thawed testicular tissues of patients with obstructive or non-obstructive azoospermia, catalase activity was higher in cryopreserved tissue of obstructive azoospermic patients suggesting that testicular tissue of such patients withstand OS induced by cryopreservation better than non-obstructive azoospermic patients [237].

The mitochondrial integrity in the testicular cells may depend upon the type of penetrating cryoprotectant used in freezing medium. Keros et al. compared the efficacy of penetrating cryoprotectants—glycerol, dimethyl sulfoxide (DMSO)—and propanediol for human testicular tissue. In spermatogonial cells, glycerol resulted in swollen mitochondria characterized by dilated cristae; propanediol resulted in heterogeneously shaped matrix in mitochondria; while DMSO preserved the morphology, which was similar to control. Based on these findings on mitochondrial integrity and cell survival, Keros et al. recommend DMSO as an ideal penetrating cryoprotectant for testicular tissue cryopreservation [238].

Studies on improving the outcome of gonadal tissue cryopreservation are limited unlike on gametes. Trehalose, a widely used non-penetrating cryoprotectant, has shown to effectively mitigate the OS in bovine calf testicular tissue during cryopreservation [236]. Ha et al., reported that addition of hypotaurine to DMSO-based freezing medium significantly improves the post-thaw mitochondrial activity in mouse spermatogonial stem cells (SSCs) [239]. Supplementing vitamin E to the mice prepubertal testicular tissue cryopreservation medium reduced the OS and improved the in vitro sperm production [235]. Similarly, in ovarian tissue, Fabbri et al., demonstrated better survival of oocytes and mitochondrial function in ovaries frozen with 30% human serum compared to 20% human serum as well as 20% fetal calf serum [240]. In a study conducted on human ovarian tissue, Talevi et al., observed that replacing sodium with choline in slow cryopreservation medium not only helps in eliminating the risk of sodium toxicity on oocytes, but it also significantly decreases the mitochondrial ultrastructural damage. In addition, stromal cells in ovarian tissue were better preserved [228]. However, to ascertain the beneficial role of using antioxidants or free radical scavengers in freezing medium of gonadal tissues needs to be validated by further studies to assess their ability to give rise to an oocyte or spermatozoa under in vitro conditions and their ability to regenerate after transplantation.

## 8. Concluding Remarks

Development and refinement of cryopreservation procedures have been fundamental in human and animal assisted reproduction and fertility preservation. However, depending on the species and the quality of gametes, cryopreservation can decrease sperm and oocytes’ performance and, ultimately, the dynamics of fertilization and embryo development.

Although evidence demonstrates that mitochondrial dysfunction and OS are two common consequences arising from cryopreservation of somatic and reproductive cells, systematic studies addressing the mechanisms inducing such damage in gametes are limited. Herein, based on literature reviewed, we propose hypothetical mechanisms of cryopreservation-associated mitochondrial dysfunction in gametes. Ca^2+^ overload induced by permeating cryoprotectants and increased ROS could cause mPTP prolonged openings triggering further ROS generation, rupture of mitochondrial membranes, Cyt C release and eventually leading to cell death. Mitochondrial sub-lethal damage in thawed or warmed cryopreserved reproductive cells can reduce their performance according to the roles played by mitochondria in sperm, oocytes, and gonadal tissues.

Overwhelming evidence indicates that treatment with a wide range of antioxidants and other means, before and during cryopreservation or after thawing/warming, can prevent or reduce mitochondrial dysfunction and OS thereby improving the reproductive potential. However, systematic comparative studies on the differential protective effects of antioxidants on cryopreserved reproductive cells are seldom present in the literature. Several studies show that mitochondrial antioxidants can ameliorate the cryopreservation outcome, whereas fewer studies have been made with mitochondria-targeted antioxidants. Despite encouraging findings on the efficacy of several antioxidants and other treatments in the prevention of mitochondrial cryoinjuries and OS, further studies are warranted before their introduction in the clinical setting. Finally, as mitochondrial ROS drive specific signaling events in reproductive cells, the careless use of antioxidant type, concentration and exposure time may reduce or ablate mitochondrial or cytoplasmic ROS below physiological thresholds leading to a reductive stress and the consequent deregulation of ROS-induced signaling.
